# Supplementary figures and images for: Efficacy and safety of Brolucizumab for neovascular age-related macular degeneration: a systematic review and meta-analysis
Source: PeerJ. 2024 Jun 21;12:e17561. doi: 10.7717/peerj.17561 (PMC11195547; doi:10.7717/peerj.17561)

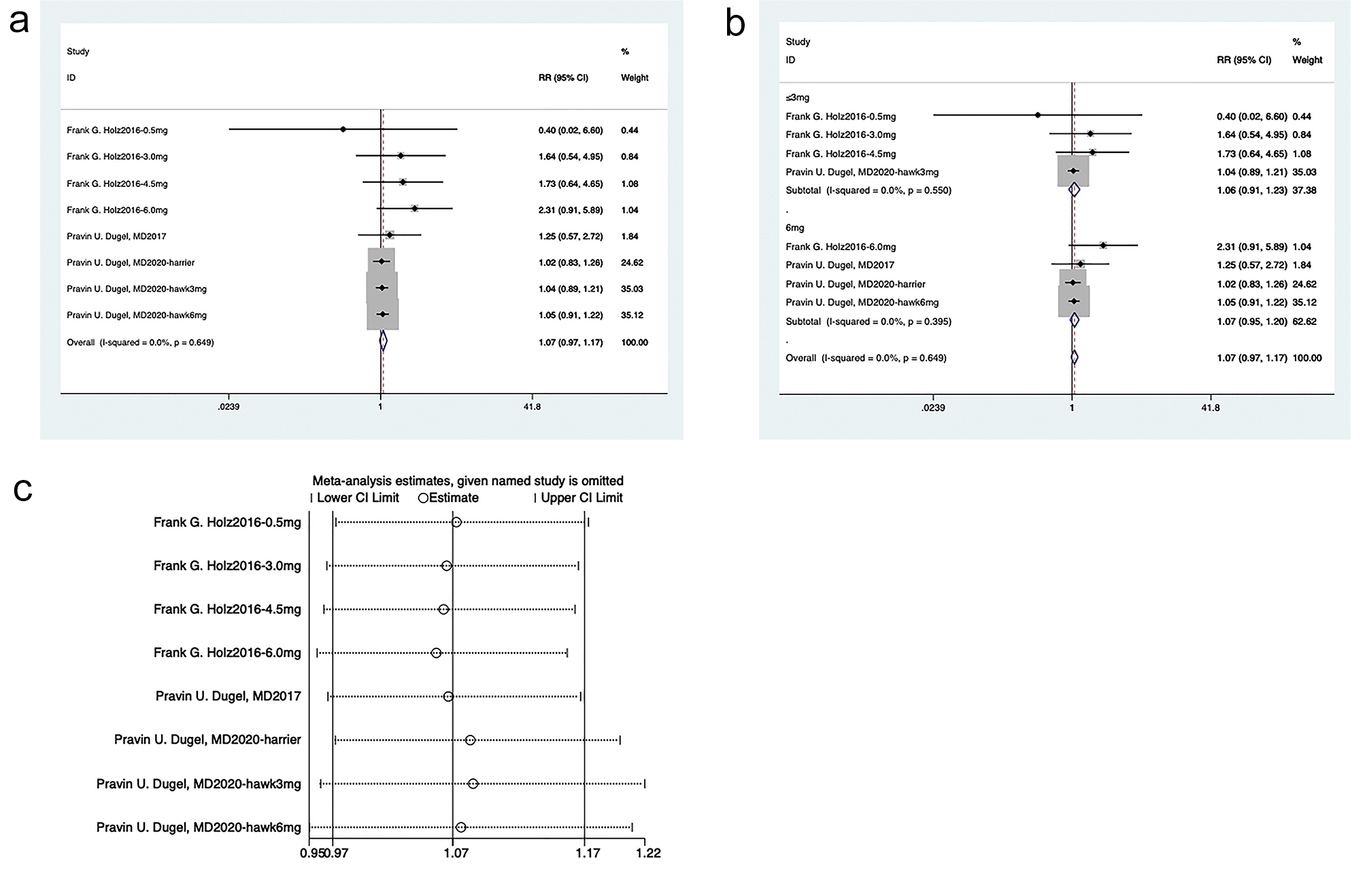

Supplement: Supplemental Information 1 [file peerj-12-17561-s001.png]

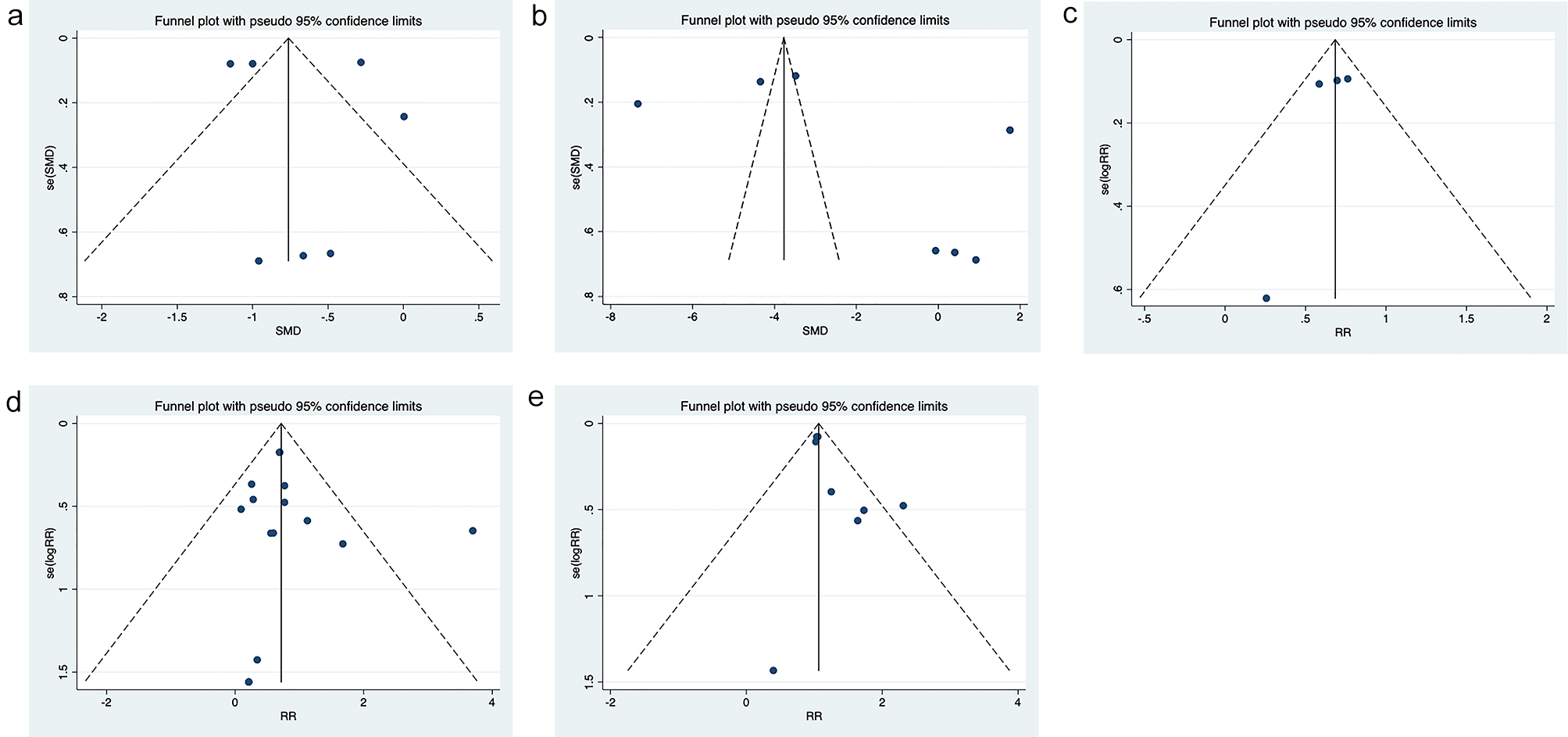

Supplement: Supplemental Information 2 [file peerj-12-17561-s002.png]
