# Supplementary material for: Efficacy and safety of Brolucizumab for neovascular age-related macular degeneration: a systematic review and meta-analysis
Source: PeerJ. 2024 Jun 21;12:e17561. doi: 10.7717/peerj.17561 (PMC11195547; doi:10.7717/peerj.17561)
Supplement: Supplemental Information 3 [file peerj-12-17561-s003.docx]

**TableS1.** PubMed Search History

| Search number | Query | Sort By | Filters | Search Details | Results | Time |
| --- | --- | --- | --- | --- | --- | --- |
| 7 | (("brolucizumab" [Supplementary Concept]) OR ((((RTH258[Title/Abstract]) OR (RTH-258[Title/Abstract])) OR (ESBA1008[Title/Abstract])) OR (ESBA-1008[Title/Abstract]))) AND (("Macular Degeneration"[Mesh]) OR (((((((((((((((((((Degeneration, Macular[Title/Abstract]) OR (Macular Degenerations[Title/Abstract])) OR (Maculopathy[Title/Abstract])) OR (Maculopathies[Title/Abstract])) OR (Macular Dystrophy[Title/Abstract])) OR (Dystrophy, Macular[Title/Abstract])) OR (Macular Dystrophies[Title/Abstract])) OR (Age-Related Macular Degeneration[Title/Abstract])) OR (Age Related Macular Degeneration[Title/Abstract])) OR (Age-Related Macular Degenerations[Title/Abstract])) OR (Macular Degeneration, Age-Related[Title/Abstract])) OR (Macular Degeneration, Age Related[Title/Abstract])) OR (Maculopathies, Age-Related[Title/Abstract])) OR (Maculopathy, Age-Related[Title/Abstract])) OR (Maculopathy, Age Related[Title/Abstract])) OR (Age-Related Maculopathies[Title/Abstract])) OR (Age Related Maculopathies[Title/Abstract])) OR (Age-Related Maculopathy[Title/Abstract])) OR (Age Related Maculopathy[Title/Abstract]))) |  |  | ("brolucizumab"[Supplementary Concept] OR ("RTH258"[Title/Abstract] OR "RTH-258"[Title/Abstract] OR "ESBA1008"[Title/Abstract] OR "ESBA-1008"[Title/Abstract])) AND ("Macular Degeneration"[MeSH Terms] OR ("degeneration macular"[Title/Abstract] OR "macular degenerations"[Title/Abstract] OR "Maculopathy"[Title/Abstract] OR "Maculopathies"[Title/Abstract] OR "macular dystrophy"[Title/Abstract] OR "dystrophy macular"[Title/Abstract] OR "macular dystrophies"[Title/Abstract] OR "age related macular degeneration"[Title/Abstract] OR "age related macular degeneration"[Title/Abstract] OR "age related macular degenerations"[Title/Abstract] OR "macular degeneration age related"[Title/Abstract] OR "macular degeneration age related"[Title/Abstract] OR "maculopathies age related"[Title/Abstract] OR "maculopathy age related"[Title/Abstract] OR "maculopathy age related"[Title/Abstract] OR "age related maculopathies"[Title/Abstract] OR "age related maculopathies"[Title/Abstract] OR "age related maculopathy"[Title/Abstract] OR "age related maculopathy"[Title/Abstract])) | 49 | 3:42:38 |
| 6 | ("Macular Degeneration"[Mesh]) OR (((((((((((((((((((Degeneration, Macular[Title/Abstract]) OR (Macular Degenerations[Title/Abstract])) OR (Maculopathy[Title/Abstract])) OR (Maculopathies[Title/Abstract])) OR (Macular Dystrophy[Title/Abstract])) OR (Dystrophy, Macular[Title/Abstract])) OR (Macular Dystrophies[Title/Abstract])) OR (Age-Related Macular Degeneration[Title/Abstract])) OR (Age Related Macular Degeneration[Title/Abstract])) OR (Age-Related Macular Degenerations[Title/Abstract])) OR (Macular Degeneration, Age-Related[Title/Abstract])) OR (Macular Degeneration, Age Related[Title/Abstract])) OR (Maculopathies, Age-Related[Title/Abstract])) OR (Maculopathy, Age-Related[Title/Abstract])) OR (Maculopathy, Age Related[Title/Abstract])) OR (Age-Related Maculopathies[Title/Abstract])) OR (Age Related Maculopathies[Title/Abstract])) OR (Age-Related Maculopathy[Title/Abstract])) OR (Age Related Maculopathy[Title/Abstract])) |  |  | "Macular Degeneration"[MeSH Terms] OR "degeneration macular"[Title/Abstract] OR "macular degenerations"[Title/Abstract] OR "Maculopathy"[Title/Abstract] OR "Maculopathies"[Title/Abstract] OR "macular dystrophy"[Title/Abstract] OR "dystrophy macular"[Title/Abstract] OR "macular dystrophies"[Title/Abstract] OR "age related macular degeneration"[Title/Abstract] OR "age related macular degeneration"[Title/Abstract] OR "age related macular degenerations"[Title/Abstract] OR "macular degeneration age related"[Title/Abstract] OR "macular degeneration age related"[Title/Abstract] OR "maculopathies age related"[Title/Abstract] OR "maculopathy age related"[Title/Abstract] OR "maculopathy age related"[Title/Abstract] OR "age related maculopathies"[Title/Abstract] OR "age related maculopathies"[Title/Abstract] OR "age related maculopathy"[Title/Abstract] OR "age related maculopathy"[Title/Abstract] | 39,172 | 3:42:22 |
| 5 | ((((((((((((((((((Degeneration, Macular[Title/Abstract]) OR (Macular Degenerations[Title/Abstract])) OR (Maculopathy[Title/Abstract])) OR (Maculopathies[Title/Abstract])) OR (Macular Dystrophy[Title/Abstract])) OR (Dystrophy, Macular[Title/Abstract])) OR (Macular Dystrophies[Title/Abstract])) OR (Age-Related Macular Degeneration[Title/Abstract])) OR (Age Related Macular Degeneration[Title/Abstract])) OR (Age-Related Macular Degenerations[Title/Abstract])) OR (Macular Degeneration, Age-Related[Title/Abstract])) OR (Macular Degeneration, Age Related[Title/Abstract])) OR (Maculopathies, Age-Related[Title/Abstract])) OR (Maculopathy, Age-Related[Title/Abstract])) OR (Maculopathy, Age Related[Title/Abstract])) OR (Age-Related Maculopathies[Title/Abstract])) OR (Age Related Maculopathies[Title/Abstract])) OR (Age-Related Maculopathy[Title/Abstract])) OR (Age Related Maculopathy[Title/Abstract]) |  |  | "degeneration macular"[Title/Abstract] OR "macular degenerations"[Title/Abstract] OR "Maculopathy"[Title/Abstract] OR "Maculopathies"[Title/Abstract] OR "macular dystrophy"[Title/Abstract] OR "dystrophy macular"[Title/Abstract] OR "macular dystrophies"[Title/Abstract] OR "age related macular degeneration"[Title/Abstract] OR "age related macular degeneration"[Title/Abstract] OR "age related macular degenerations"[Title/Abstract] OR "macular degeneration age related"[Title/Abstract] OR "macular degeneration age related"[Title/Abstract] OR "maculopathies age related"[Title/Abstract] OR "maculopathy age related"[Title/Abstract] OR "maculopathy age related"[Title/Abstract] OR "age related maculopathies"[Title/Abstract] OR "age related maculopathies"[Title/Abstract] OR "age related maculopathy"[Title/Abstract] OR "age related maculopathy"[Title/Abstract] | 26,534 | 3:40:46 |
| 4 | "Macular Degeneration"[Mesh] | Most Recent |  | "Macular Degeneration"[MeSH Terms] | 27,748 | 3:36:34 |
| 3 | ("brolucizumab" [Supplementary Concept]) OR ((((RTH258[Title/Abstract]) OR (RTH-258[Title/Abstract])) OR (ESBA1008[Title/Abstract])) OR (ESBA-1008[Title/Abstract])) |  |  | "brolucizumab"[Supplementary Concept] OR "RTH258"[Title/Abstract] OR "RTH-258"[Title/Abstract] OR "ESBA1008"[Title/Abstract] OR "ESBA-1008"[Title/Abstract] | 76 | 3:25:21 |
| 2 | (((RTH258[Title/Abstract]) OR (RTH-258[Title/Abstract])) OR (ESBA1008[Title/Abstract])) OR (ESBA-1008[Title/Abstract]) |  |  | "RTH258"[Title/Abstract] OR "RTH-258"[Title/Abstract] OR "ESBA1008"[Title/Abstract] OR "ESBA-1008"[Title/Abstract] | 5 | 3:15:53 |
| 1 | "brolucizumab" [Supplementary Concept] | Most Recent |  | "brolucizumab"[Supplementary Concept] | 73 | 3:14:18 |
