# Supplementary material for: Efficacy and safety of Brolucizumab for neovascular age-related macular degeneration: a systematic review and meta-analysis
Source: PeerJ. 2024 Jun 21;12:e17561. doi: 10.7717/peerj.17561 (PMC11195547; doi:10.7717/peerj.17561)
Supplement: Supplemental Information 4 [file peerj-12-17561-s004.docx]

## Systematic Review and/or Meta-Analysis Rationale

For systematic reviews / meta-analyses, authors need to provide the following information:

1.The rationale for conducting the systematic review / meta-analysis.

**Reply:** A network meta-analysis compared the efficacy of different VEGF monoclonal antibodies in the treatment of nAMD, and Brolucizumab showed a more prominent effect. In addition, Brolucizumab has been shown to be the result of switching to brolucizumab in patients with neovascular age-related macular degeneration with an incomplete response to Ranibizumab or Aflibercept.

2.The contribution that it makes to knowledge in light of previously published related reports, including other meta-analyses and systematic reviews.

**Reply:** Although there have been mesh meta-analyses comparing the efficacy of Brolucizumab with other anti-VEGF drugs, no meta-analysis has been published on the safety and efficacy of Brolucizumab, and recent studies have shown that there are certain adverse events associated with the use of Brolucizumab.
